# Brain Plasticity in Charcot-Marie-Tooth Type 1A Patients? A Combined Structural and Diffusion MRI Study

**DOI:** 10.3389/fneur.2020.00795

**Published:** 2020-09-08

**Authors:** Giuseppe Pontillo, Raffaele Dubbioso, Sirio Cocozza, Stefano Tozza, Daniele Severi, Rosa Iodice, Enrico Tedeschi, Andrea Elefante, Arturo Brunetti, Fiore Manganelli, Mario Quarantelli

**Affiliations:** ^1^Department of Advanced Biomedical Sciences, University Federico II, Naples, Italy; ^2^Department of Neurosciences, Reproductive and Odontostomatological Sciences, University Federico II, Naples, Italy; ^3^Institute of Biostructure and Bioimaging, National Research Council, Naples, Italy

**Keywords:** Charcot-Marie-Tooth disease, structural magnetic resonance imaging, diffusion magnetic resonance imaging, cerebellum, peripheral nervous system diseases, electrophysiology

## Abstract

Central nervous system involvement has been described in peripheral neuropathies, including different forms of Charcot-Marie-Tooth (CMT) disease. The aim of our study was to systematically investigate possible brain structural modifications in CMT1A patients, using volumetric MRI, and diffusion tensor imaging (DTI). In this prospective cross-sectional study, from May 2017 to May 2019, we acquired 3T MRI brain scans of genetically confirmed CMT1A patients and age- and sex-comparable healthy controls. Patients also underwent clinical and electrophysiological examinations assessing motor and sensory domains. Voxel-based morphometry (VBM) and tract-based spatial statistics (TBSS) analyses were performed using a non-parametric approach based on permutations, including age and sex (and total intracranial volume for VBM) as nuisance covariates. When between-group differences emerged at VBM or TBSS analyses, the first eigenvariate was extracted from the cluster and its age- and sex-adjusted standardized residuals tested for correlation with clinical and electrophysiological variables. Twenty CMT1A patients (34.5 ± 11.1 years; M/F:11/9) were enrolled, along with 20 healthy controls (30.1 ± 10.2 years; M/F:11/9). The VBM analysis revealed clusters of significantly increased GM volume in CMT1A patients compared to healthy controls, encompassing the bilateral cerebellar lobules III-VI and the left hippocampus (all *p*s = 0.04), with no differences in terms of DTI metrics at the TBSS analysis. A negative correlation (*r* = −0.502, *p* = 0.03) emerged between ulnar compound motor action potential and the *z*-scores corresponding to the right cerebellar cluster of augmented GM volume. Our data show evidence of structural reorganization in the brain of CMT1A patients, possibly reflecting neural plasticity mechanisms in response to peripheral nerve pathology and modulating the effect of axonal degeneration on functional impairment.

## Introduction

Charcot-Marie-Tooth disease (CMT) consists of a clinically and genetically heterogeneous group of inherited peripheral neuropathies, representing the most frequent hereditary neuromuscular disorders, with a reported prevalence of 1:2500 (1). The most common form of CMT is the type 1A (CMT1A, MIM#118220), an autosomal dominant demyelinating neuropathy caused by a duplication of the peripheral myelin protein 22 (*PMP22*) gene located on chromosome 17, representing about 80% of all demyelinating forms of CMT (2). It usually presents as the “classical CMT” phenotype, characterized, within the first two decades, with the onset of difficulty with walking, sensory loss, foot deformities, and signs of a length dependent sensorimotor neuropathy (3).

Although CMT is primarily a peripheral nervous system disease, several cases of central nervous system (CNS) involvement have been described in different forms of CMT (4, 5), including patients with *PMP22* duplication (6, 7). Along with these anecdotal reports, a previous study reported reduced cerebral white matter (WM) global volume in patients with *PMP22* deletion/duplication (8), while WM microstructural abnormalities have been recently demonstrated in a genetically heterogeneous group of CMT patients, even though no significant regional WM damage was found in CMT1A disease (9).

Furthermore, it has been proven that peripheral nervous system pathology can induce secondary functional and structural changes in the CNS, as shown by different evidence from other peripheral neuropathies (10–12).

Given this background, the aim of our study was to investigate possible gray matter (GM) and WM structural modifications in a homogeneous group of genetically defined CMT1A patients, using volumetric MRI and diffusion tensor imaging (DTI), respectively. In addition, we explored the possible functional impact of these changes, correlating MRI findings with clinical and electrophysiological measures.

## Materials and Methods

### Subjects

In this observational cross-sectional study, from May 2017 to May 2019, we enrolled symptomatic patients with genetically confirmed *PMP22* duplication (3) along with a group of age- and sex-comparable healthy controls (HC). Exclusion criteria included age <18 years and the presence of other relevant neurological, psychiatric, or systemic conditions that could affect peripheral nerves or CNS.

The study was conducted in compliance with the ethical standard and approved by the local Ethics Committee (#100/17). Written informed consent was obtained from all subjects according to the Declaration of Helsinki.

### Clinical and Electrophysiological Evaluation

On the same day of the MRI exam, CMT1A patients underwent clinical and electrophysiological examinations mainly oriented toward the assessment of motor and sensory domains, with the following protocol:

- Charcot-Marie-Tooth Neuropathy Score (CMTNS, second version) (13), considered as a global measure of clinical disability and defined as the sum of two distinct sub scores: the CMT examination score (CMTES), rating the patients' symptoms and signs, and the CMTNS neurophysiological component, based on the assessment of ulnar compound motor action potential (CMAP) and radial sensory action potential (SAP) on the non-dominant side as objective indexes of peripheral axonal damage. The total score ranges from 0 (no disability) to 36 (maximum disability);- A Short Form-36 (SF-36) questionnaire, divided into Mental (SF36_Mental) and Physical (SF36_Physical) functions, was used to evaluate quality of life (14);
- Maximal Voluntary Isometric Contraction (MVIC) with a hand-held myometer to measure hand-grip and three-point pinch strength (15);
- 9-Hole Peg Test (9HPT) of both dominant and non-dominant sides to assess manual dexterity (16).
- 10-Meter Walk Test (10MWT) (16) and 6-Min Walk Test (6MWT) (17) for walking ability.

### MRI Data Acquisition

All MRI exams were performed on the same 3-T scanner (Trio, Siemens Medical Systems, Erlangen, Germany), with the acquisition protocol including a structural T1-weighted volume acquired using a 3D Magnetization Prepared Rapid Acquisition Gradient Echo sequence (MPRAGE; TR = 2,300 ms; TE = 2,96 ms; TI = 1,100 ms; Flip Angle = 9°; voxel size = 1 × 1 × 1 mm^3^; 192 sagittal slices) for the voxel-based morphometry (VBM) analysis (18), along with a DTI dataset acquired using an echo-planar imaging sequence (TR = 7,400 ms; TE = 88 ms, 64 directions uniformly distributed in three dimensional space; B-factors 0 and 1,000 s/mm^2^, 9 B0 images equally spaced throughout the DTI acquisition, voxel size = 2.2 × 2.2 × 2.2 mm^3^, 60axial slices) for the tract-based spatial statistics (TBSS) analysis (19) and a 2D T2-weighted Fluid Attenuated Inversion Recovery (FLAIR; TR = 8,500 ms; TE = 106 ms; TI = 2,500 ms; Flip Angle = 150°; voxel size = 0.9 × 0.9 × 4 mm^3^; 25 axial slices) sequence for possible incidental lesions detection.

### MRI Data Analysis

Before image processing, an experienced radiologist with more than 20 years of practice in the field of neuroimaging (MQ) preliminarily inspected acquired scans to check image quality and exclude the presence of incidental lesions or malformations.

For the VBM analysis, structural data were processed using the Statistical Parametric Mapping software package (SPM12, http://www.fil.ion.ucl.ac.uk/spm) via the Computational Anatomy Toolbox (CAT12, http://www.neuro.uni-jena.de/cat) in Matlab R2019a (The Mathworks, Inc., Natick, MA, USA). We used the default settings, described in detail in the CAT12 manual (http://dbm.neuro.uni-jena.de/cat12/CAT12-Manual.pdf). Pre-processing steps included spatial registration of T1-weighted volumes to a reference brain template in Montreal Neurological Institute (MNI) space using a fast diffeomorphic registration algorithm (Diffeomorphic Anatomical Registration using Exponentiated Lie algebra, DARTEL) (20), tissue segmentation in GM, WM, and cerebrospinal fluid (CSF) and bias correction of intensity non-uniformities. Normalized GM maps were then modulated by scaling by the inverse of the amount of the volume changes due to spatial registration, in order to preserve the local GM volumes. Homogeneity of VBM data was checked using the CAT12 default function in order to identify possible outliers. Finally, normalized modulated GM images were spatially smoothed using a 1 mm Full Width at Half Maximum isotropic Gaussian kernel (21). The same procedure was also applied to normalized WM maps.

For each participant, the Total Intracranial Volume (TIV) was also estimated using the standard procedure implemented in CAT12 and used as confound in subsequent statistical analyses in order to correct for individual head sizes.

TBSS analysis was performed using FSL v6.0 (FMRIB's Software Library, http://fsl.fmrib.ox.ac.uk/fsl). DTI data were preliminarily corrected for head motion and eddy current distortions using eddy_correct (22), and diffusion gradient directions were adjusted according to the corresponding deformation vectors (23). Subsequently, for each study a brain mask was obtained from B0 images using the Brain Extraction Tool (24) and a tensor model was fitted to diffusion data to generate fractional anisotropy (FA), mean diffusivity (MD), axial diffusivity (AD), and radial diffusivity (RD). All subjects' FA volumes were then aligned to a common target in the MNI space (FMRIB58_FA) using the non-linear registration tool (FNIRT) and interpolated to a voxel size of 1 × 1 × 1 mm^3^. Next, the mean FA image was created and thinned to create a mean FA skeleton representing the centers of all tracts common to the group. Finally, each subject's aligned FA maps were projected onto this skeleton for the statistical analysis. Similarly, the FA-derived non-linear registrations and FA skeleton were used for the processing of other non-FA diffusion metrics (i.e., MD, AD, and RD).

### Statistical Analysis

Unless otherwise specified, statistical analyses were carried out using the Statistical Package for Social Science (SPSS v24.0, IBM corp., Armonk, NY), with a statistical significance threshold set at *p* < 0.05, and the Benjamini-Hochberg procedure was adopted for controlling the false discovery rate (FDR) (25).

Differences between CMT1A patients and HC in terms of age, sex, and handedness were tested using Student *t*-test (age) and Fisher's exact test (sex and handedness), while possible alterations of GM, WM, and CSF volume fractions (GMf, WMf, and CSFf–defined as the ratio to TIV of GM, WM, and CSF volumes, respectively), were assessed with ANCOVA analyses, correcting for age, and sex.

For the VBM analysis, the normalized, modulated, and smoothed GM and WM maps were statistically analyzed to assess local volume differences between the two groups using a non-parametric approach based on permutations applied to the general linear model (26) via SPM's Threshold Free Cluster Enhancement (TFCE) toolbox (http://www.neuro.uni-jena.de/tfce), including age, sex, and TIV as confounding variables. Using the TFCE approach (19), 5,000 permutations were generated and cluster-like structures were enhanced, with a significance level set at *p* < 0.05, corrected for multiple comparisons across space using the FDR method (*q* < 0.05) (25). The only voxels considered significant were part of a spatially continuous cluster size of 100 isotropic voxels or more.

For the TBSS, skeletonized FA maps were fed into a mirror voxel-wise cross-subject non-parametric analysis using randomize v2.9 (included in FSL v6.0) (26) with 5,000 permutations, including age and sex as nuisance covariates. As in the VBM analysis, a TFCE approach was adopted, with a statistical significance threshold set at *q* < 0.05. Likewise, the same statistical procedure was run on skeletonized non-FA maps.

For all between-group analyses, both contrasts (i.e., HC > CMT and HC < CMT) were tested.

In order to compare the magnitude of differences between groups, we computed effect sizes for statistically significant clusters' peaks using Cohen's *d* [*d* = 2 t/sqrt(df)] (27).

When regional differences in terms of GM or WM volume or DTI metrics emerged between the two groups, the corresponding first eigenvariate was extracted from the cluster and adjusted for age, sex, and TIV (the latter for the VBM clusters only). The so obtained residuals were standardized and their relationship with clinical and electrophysiological variables was assessed via robust Pearson correlation analyses, using bootstrap with 1,000 replications. Correlations were not adjusted for multiple testing given the exploratory nature of the analyses (28).

## Results

### Subjects

Twenty CMT1A patients (34.5 ± 11.1 years; M/F: 11/9) were enrolled, along with 20 HC of comparable age and sex (30.1 ± 10.2 years; M/F: 11/9).

Demographics of the study population are presented in Table 1, while results of the clinical and electrophysiological evaluations are depicted in Table 2.

**Table 1:** Demographics and global brain volumes of all the subjects included in the study.

	**CMT1A**	**HC**	***p*-value (test statistic)**
Number	20	20	–
Age (years)	34.5 ± 11.1	30.1 ± 10.2	0.20 (1.305)^*^
Sex	11M/9F	11M/9F	1.00 (0.000)^**^
Hand dominance	18R/2L	20R	0.49 (2.105)^**^
GMf (%)	46.7 ± 2.2	46.3 ± 3.1	0.62 (0.250)^***^
WMf (%)	35.3 ± 1.5	36.5 ± 2.0	0.04 (4.569)^***^^#^
CSFf (%)	18.0 ± 2.9	17.1 ± 2.8	0.047 (4.225)^***^^#^

* *T-value*,

** *Pearson Chi-Square value*,

*** *F-value (2, 36)*,

# *not significant after multiple testing correction*.

*GMf, Gray Matter fraction; WMf, White Matter fraction; CSFf, Cerebro-Spinal Fluid fraction*.

**Table 2:** Results of the clinical and neurophysiological examinations of CMT1A patients.

	**CMT1A**	
	***N***	**Mean (SD)**
CMTNS		
Total score	18	9.9 (3.7)
CMTES	20	5.1 (2.9)
Neurophysiological component	18	4.4 (1.5)
MVIC (*N*, average for both sides)		
Handgrip	20	74.7 (22.9)
Three-point pinch	20	67.7 (25.2)
9-HPT (s, average for both sides)	20	23.9 (3.8)
10MWT (s)	20	6.8 (1.5)
6MWT (m)	20	428.8 (70.2)
SF-36		
Physical composite score	20	60.0 (21.1)
Mental composite score	20	68.3 (18.0)
Ulnar CMAP (mV)	18	4.9 (1.6)
Radial SAP (μV)	18	1.4 (2.4)

*CMT, Charcot-Marie-Tooth; HC, Healthy Controls; CMTNS, Charcot-Marie-Tooth Neuropathy Score; CMTES, Charcot-Marie-Tooth Examination Score; MVIC, Maximal Voluntary Isometric Contraction; 9HPT, 9-Hole Peg Test; 10MWT, 10-Meter Walk Test; 6MWT, 6-Min Walking Test; SF-36, Short Form (36); CMAP, Compound Muscle Action Potential; SAP, Sensory Action Potential*.

We found decreased WMf (Cohen's *d* = 0.71, *p* = 0.04, not significant after multiple testing correction) in CMT1A patients compared to HC, along with an increase in CSFf (Cohen's *d* = 0.69*, p* = 0.047, not significant after multiple testing correction), while no significant difference in terms of global GMf emerged (Table 1). As expected, age was negatively related to GMf (β = −0.002, *p* < 0.001) and positively related to CSFf (β = 0.002, *p* < 0.001), while no significant relationships emerged between age and WMf or between the other covariate (i.e., sex) and either GM, WM, or CSF fractions.

### VBM and TBSS Analyses

**Figure 1:**
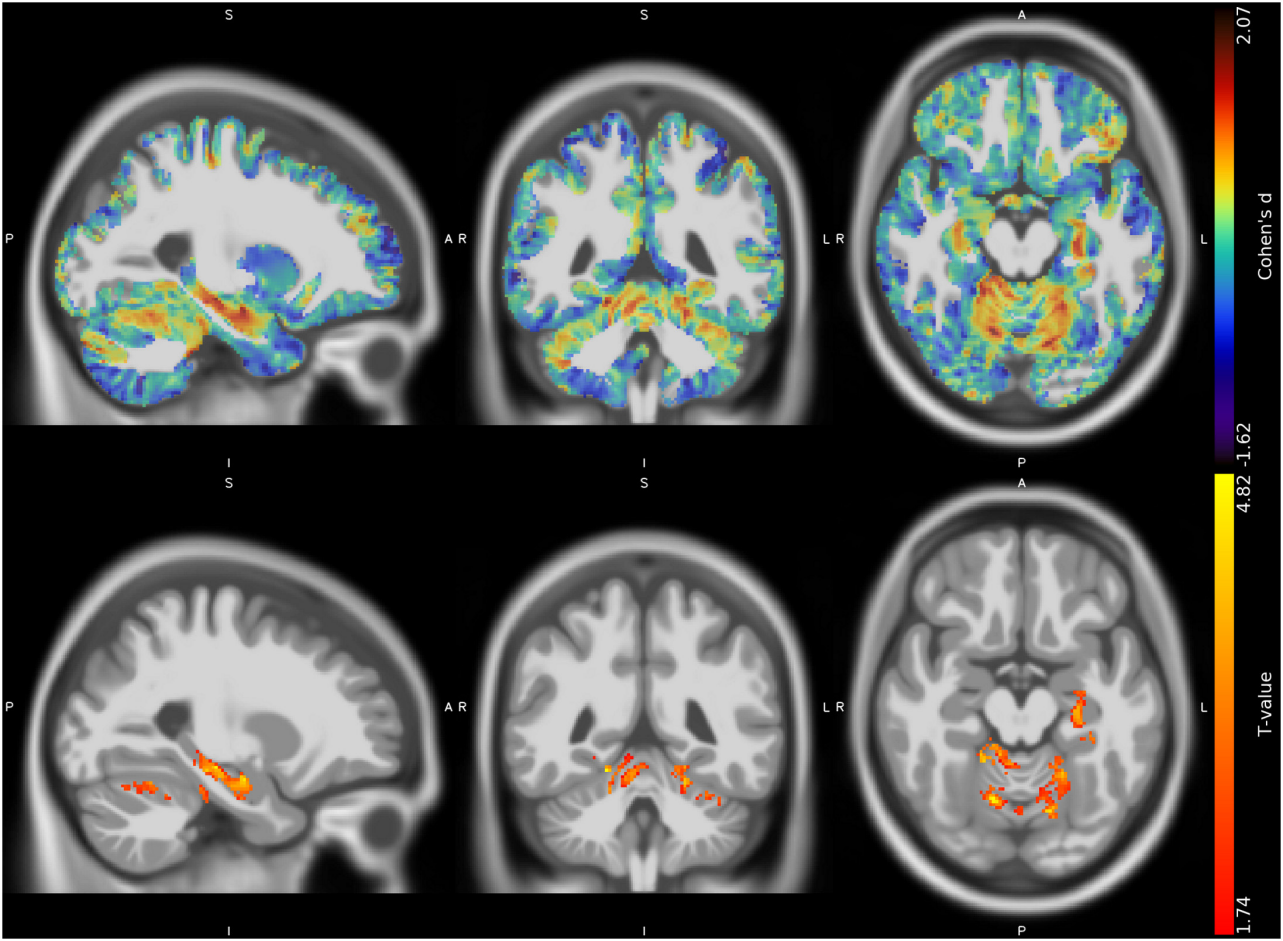
Unthresholded effect size (Cohen's *d*) map (*upper row, in blue-red*) and thresholded T map (*lower row, in red-yellow*) for the CMT > HC contrast regarding the voxel-based between-group comparison of GM maps, superimposed on the T1-weighted template in the sagittal, coronal, and axial planes (*from left to right*). CMT, Charcot-Marie-Tooth; HC, Healthy Controls; GM, Gray Matter.

The VBM analysis revealed three different clusters of significantly increased GM volume in CMT1A patients compared to HC encompassing the right paravermian portions of the cerebellar lobules III, IV, V, and VI (*p* = 0.04, *d* = 1.62), lobules IV, V, and VI of the contralateral cerebellum (*p* = 0.04, *d* = 1.63) and the left hippocampus and parahippocampal region (*p* = 0.04, *d* = 1.61; Figure 1, Table 3).

**Table 3:** Clusters of increased GM volume in CMT patients compared to HC are presented, along with significance level (FDR-corrected) and the corresponding local maxima's effect sizes, *T*-values, and anatomical labels.

**Cluster volume (ml)**	***p*-value (FDR-corr)**	**Cohen's *d***	**T**	**MNI coordinates (mm)**			**Anatomical label**
				**X**	**Y**	**Z**	
2.63	0.04	1.62	4.80	14	−63	−15	Right cerebellar lobule VI
	0.04	1.59	4.71	14	−39	−18	Right cerebellar lobule III
	0.04	1.62	4.78	18	−47	−14	Right cerebellar lobules IV-V
2.13	0.04	1.61	4.78	−26	−24	−12	Left hippocampus
	0.04	1.23	3.64	−30	−30	−18	Left parahippocampus
2.74	0.04	1.48	4.37	−21	−51	−18	Left cerebellar lobules IV-V
	0.04	1.63	4.82	−12	−69	−17	Left cerebellar lobule VI

*No significant differences emerged when testing the HC > CMT contrast. Coordinates refer to mm from the anterior commissure in MNI space, with anatomical labeling according to (29)*.

*DF = 35*.

*GM, Gray Matter; CMT, Charcot-Marie-Tooth; HC, Healthy Controls; FDR, False Discovery Rate; MNI, Montreal Neurological Institute; DF, Degrees of Freedom*.

**Figure 2:**
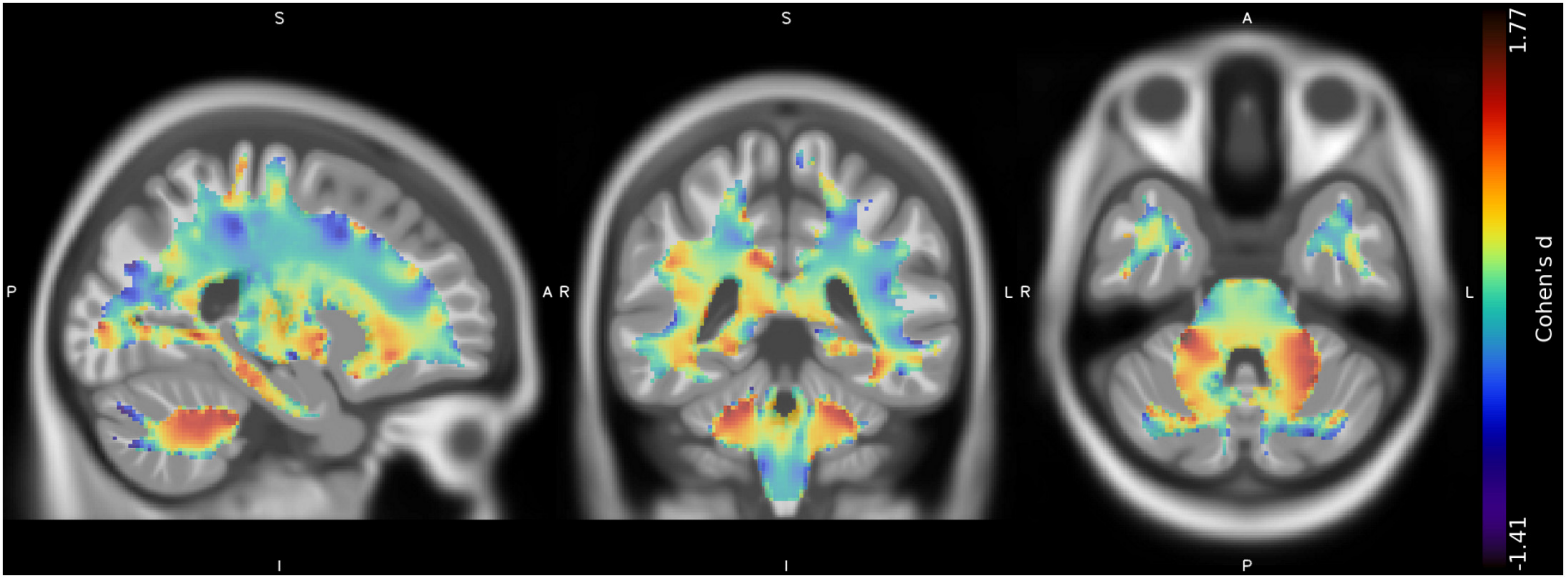
Unthresholded effect size (Cohen's *d*) map (*in blue-red*) for the CMT > HC contrast regarding the voxel-based between-group comparison of WM maps, superimposed on the T1-weighted template in the coronal and axial planes (*from left to right*). CMT, Charcot-Marie-Tooth; HC, Healthy Controls; WM, White Matter.

No suprathreshold clusters of altered WM quantity in CMT1A compared to HC emerged, even if the effect size map showed conspicuous areas of increased volume in the bilateral superior cerebellar WM (Figure 2), not reaching statistical significance.

Given the prominence of cerebellar findings emerging from the VBM investigation, we decided to validate these results in a *post hoc* analysis by means of a cerebellum-tailored approach using the Spatially Unbiased Infratentorial Toolbox (SUIT) version 3.4, implemented in SPM12. Cerebellar analysis substantially confirmed the results of the whole-brain investigation, showing clusters of increased GM volume in CMT1A patients compared to HC encompassing the bilateral anterior lobe (*p* = 0.0498, *d* = 1.80), the right cerebellar lobule VI (*p* = 0.0498, *d* = 1.44), and right (*p* = 0.0498, *d* = 1.41) and left (*p* = 0.0498, *d* = 1.13) cerebellar crus I. Details regarding the methods and the results of the *post hoc* analysis are reported in the Supplementary Material.

No statistically significant between-group differences emerged at the TBSS analysis when considering the FA, MD, AD, and RD metrics.

### Relationship Between MRI Metrics and Clinical and Electrophysiological Data

**Figure 3:**
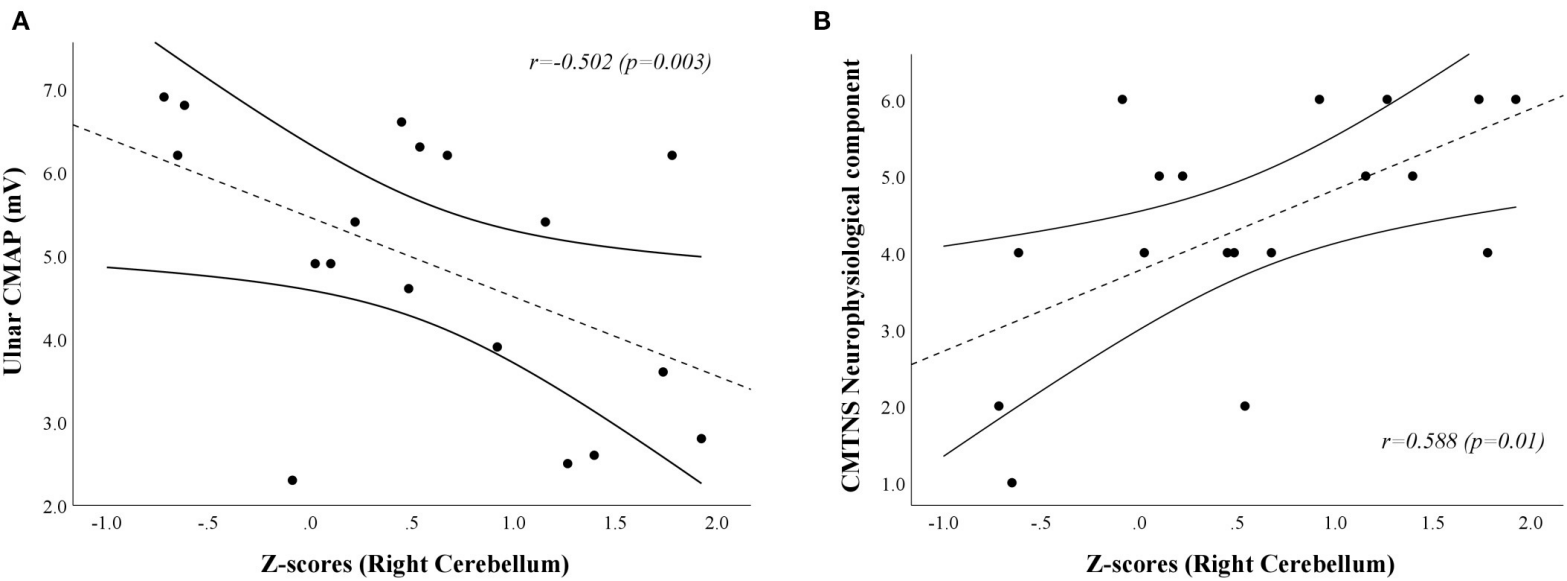
Scatterplots showing the correlation between age-, sex-, and TIV-adjusted *z*-scores extracted from the right cerebellar cluster of increased GM volume in CMT1A patients and ulnar CMAP [**(A)**; *r* = −0.502, Bca 95% CI (−0.805–−0.142), *p* = 0.03] and CMTNS neurophysiological component [**(B)**;*r* = 0.588, Bca 95% CI (0.055–0.876), *p* = 0.01] values, respectively. CMAP is expressed in mV, while the score of the CMTNS neurophysiological component is a dimensionless quantity ranging from 0 (no neurophysiological impairment) to 8 (maximum neurophysiological impairment). The dashed line represents the fitted linear model curve, while solid lines indicate the 95% confidence interval of the prediction. GM, Gray Matter; CMT, Charcot-Marie-Tooth; HC, Healthy Controls; CMTNS, Charcot-Marie-Tooth Neuropathy Score; Bca 95% CI, Bias corrected and accelerated bootstrap 95% confidence interval; CMAP, Compound Muscle Action Potential.

A positive correlation [*r* = 0.588, Bias corrected and accelerated bootstrap 95% confidence interval (0.055–0.876), *p* = 0.01] was found between the neurophysiological component of the CMTNS and the age-, sex-, and TIV-adjusted z-scores of the first eigenvariate extracted from the right cerebellar cluster of significant between-group difference at the VBM analysis, mainly resulting from a negative relationship between *z*-scores and ulnar CMAP values [*r* = −0.502, Bca 95% CI (−0.805–−0.142), *p* = 0.03; Figure 3]. No significant correlation emerged between the clusters of increased GM density and the remaining clinical and electrophysiological measures of functional impairment in CMT1A patients.

## Discussion

In this study, we investigated the presence of possible structural modifications in the brain of CMT1A patients, providing evidence of both global WM volume decrease and regional, mostly cerebellar, GM volume increase in this condition, correlating with electrophysiological measures.

Despite evidence of brain involvement in other forms of CMT (4, 5), and, more widely, in other peripheral neuropathies (10–12), CMT1A is commonly considered as a purely peripheral nervous system disease. Indeed, PMP22 is produced primarily by Schwann cells and it is expressed in the compact portion of essentially all myelinated fibers in the peripheral nervous system (3, 30). Actually, apart from anecdotal reports, little or no structured evidence exists on the involvement of CNS in these patients.

In our study, CMT1A patients showed almost significant reduction of global normalized WM volume and increase in the amount of CSF compared to HC, with no significant changes in terms of global GM volume. This result partially replicates the one of a previous study (8) demonstrating a reduction of global WM volume in *PMP22*-related neuropathies (i.e., CMT1A and hereditary neuropathy with liability to pressure palsies–HNPP). The physiopathological mechanisms behind these observed changes remain unclear, with a possible role of *PMP22* and its corresponding mRNA in the development of CNS that has been hypothesized (8). Indeed, a limited amount of PMP22 protein has been demonstrated in the normal human CNS, along with a more widespread expression of *PMP22* mRNA (31). These molecules allegedly play a role in the regulation of cell growth and differentiation (30, 31), so that an alteration of their expression, especially in early phases of neurodevelopment, may account for a disturbance of brain structural organization (8).

Additionally, the precocious and chronic reduction of the burden of afferent and efferent stimuli to and from the CNS, due to CMT1A peripheral neuropathy, may as well-influence the brain's macrostructure.

Conversely, when looking at WM microstructure, the TBSS analysis revealed no significant abnormalities in CMT1A patients compared to HC, confirming the results of a previous exploratory study conducted on a heterogeneous population of CMT patients, in which CMT1A patients proved to be the only CMT subtype not affected by brain WM microstructural damage (9). These results suggest an overall harmonic reduction of microstructurally intact fibers rather than actual WM damage in this condition.

In terms of possible regional volumetric alterations, results of the VBM analysis revealed that CMT1A patients show clusters of increased GM volume compared to HC in specular paravermian portions of the anterior cerebellum (i.e., lobules III, IV, and V) and lobule VI. Along with this clusters of statistically significant between-group difference, effect size maps also showed evident areas of increased volume in the bilateral superior cerebellar WM, not reaching statistical significance most probably due to the small sample size (32). According to the cerebellar functional topographic organization, lobules of the anterior lobe, and lobule VI contain the representation of the sensorimotor cerebellum (33), with the vermian and intermediate zones, in particular, corresponding to the more classic functional/phylogenetic definition of spinocerebellum/paleocerebellum (34). These regions, somatotopically arranged, are widely linked to spinal cord, brainstem, and cerebral cortical areas concerned with sensorimotor processing and participate in the coordination of fine movements of the extremities as well as in the maintenance of balance and gait (33, 34). Hence, in a condition characterized by long-lasting impairment of distal muscle strength, manual dexterity, and walking ability (3), as well as by reduced afferent and efferent stimuli to and from the CNS, the observed increase of anterior cerebellar regional volume might reflect mechanisms of structural plasticity (35) aimed at compensating peripheral nerve deficit and enduring brain deafferentation in CMT1A patients. Indeed, similar increases of cerebellar regional GM volume have been demonstrated in healthy individuals professionally subjected to the persistent solicitation of specific motor or cognitive abilities (36, 37), while brain structural and functional plasticity phenomena have also been reported in other peripheral neuropathies (11, 38). In accordance with this speculation, in our study we found an inverse correlation between the increase of GM volume in the right anterior cerebellum and the CMAP obtained from ulnar motor nerve, considered as a measure of distal arm axonal damage (15). It is known that axonal degeneration is the main determinant of neurological dysfunction and clinical disability in CMT1A patients (3, 39, 40). Therefore, greater peripheral nerve pathology might lead to a greater compensatory neuroplasticity effort by the anterior cerebellar GM, which modulates the effect of axonal degeneration on functional impairment, thus possibly explaining the lack of correlation between cerebellar structural modifications and clinical functional tests. If confirmed by longitudinal and functional MRI studies, this theory could provide new insights into the mechanisms of CNS modifications associated to peripheral nerve pathology, and how these participate in the genesis of neurological dysfunction.

Furthermore, we found a cluster of increased GM volume in the left hippocampus and parahippocampal cortex, which are known to play a crucial role for declarative memory (41). The hippocampus, in particular, demonstrates unique cellular and synaptic flexibility in the adult brain (35), with evidence of activity-dependent reorganization in both healthy subjects (42) and neuropsychiatric conditions (43, 44). Indeed, a slight cognitive impairment has been described in CMT1A patients, predominantly involving executive functions, working memory, and verbal episodic memory (8), along with minor depressive symptoms (8, 45), which might prompt neural plasticity in the hippocampus as a putative mechanism of resilience/compensation.

To better comprehend the physiopathology underlying these observed structural changes, and to investigate their clinical relevance, further studies are warranted focusing on functional MRI associated with a finer clinical, neuropsychological, and electrophysiological evaluation, also including the investigation of cognitive domains (primarily memory and information processing) and the functional examination of distal legs, posture, balance, and gait, which were lacking in our study. Likewise, longitudinal studies could help unravel the causal relationship between CNS functional and structural modifications, peripheral nerve pathology, and neurological dysfunction.

In conclusion, our data show evidence of structural reorganization in the brain of CMT1A patients, mostly involving the anterior cerebellum and possibly reflecting compensatory mechanisms in response to peripheral nerve pathology. These results provide new insights into CNS physiopathology and its role in the development of clinical disability in this condition.

## Data Availability Statement

The data that support the findings of this study are available from the corresponding author, upon reasonable request.

## Ethics Statement

The studies involving human participants were reviewed and approved by Ethics Committee Carlo Romano, University of Naples Federico II (#100/17). The patients/participants provided their written informed consent to participate in this study.

## Author Contributions

GP: conceptualization, data curation, formal analysis, wrote of the manuscript (original draft). RD and SC: conceptualization, data curation, wrote of the manuscript (review & editing). ST: data curation, investigation, wrote of the manuscript (review & editing). DS and RI: data curation, investigation. ET and AE: supervision, wrote of the manuscript (review & editing). AB and FM: supervision, investigation, wrote of the manuscript (review & editing). MQ: supervision, formal analysis, investigation, wrote of the manuscript (review & editing). All authors contributed to the article and approved the submitted version.

## Conflict of Interest

GP and SC received fees for speaking from Genzyme. The remaining authors declare that the research was conducted in the absence of any commercial or financial relationships that could be construed as a potential conflict of interest.

## Abbreviations

10MWT: 10-Meter Walk Test
6MWT: 6-Minute Walking Test
9HPT: 9-Hole Peg Test
CMAP: Compound Muscle Action Potential
CMT: Charcot-Marie-Tooth
CMTES: Charcot-Marie-Tooth Examination Score
CMTNS: Charcot-Marie-Tooth Neuropathy Score
CSF: Cerebro-Spinal Fluid
CSFf: Cerebro-Spinal Fluid fraction
FDR: False Discovery Rate
GMf: Gray Matter fraction
HC: Healthy Controls
MVIC: Maximal Voluntary Isometric Contraction
PMP22: Peripheral Myelin Protein 22
SAP: Sensory Action Potential
SF-36: Short Form (36)
TBSS: Tract-Based Spatial Statistics
TFCE: Threshold Free Cluster Enhancement
TIV: Total Intracranial Volume
VBM: Voxel-Based Morphometry
WMf: White Matter fraction.
