# Supplementary material for: Brain Plasticity in Charcot-Marie-Tooth Type 1A Patients? A Combined Structural and Diffusion MRI Study
Source: Front Neurol. 2020 Sep 8;11:795. doi: 10.3389/fneur.2020.00795 (PMC7506188; doi:10.3389/fneur.2020.00795)
Supplement: Supplementary file 1 [file Data_Sheet_1.PDF]

BRAIN PLASTICITY IN CHARCOT-MARIE-TOOTH TYPE 1A PATIENTS? A COMBINED STRUCTURAL AND DIFFUSION MRI STUDY

POST HOC ANALYSIS

Methods

As a post hoc analysis, a cerebellum-tailored voxel-based morphometry (VBM) investigation was performed using the Spatially Unbiased Infratentorial Toolbox (SUIT) version 3.4 (Diedrichsen, 2006), implemented in the Statistical Parametric Mapping (SPM12) software (http://www.fil.ion.ucl.ac.uk/spm), as described in previous works (Cocozza et al., 2017).

Briefly, for each subject, the cerebellum was automatically identified and isolated on 3D-T1w images to obtain a cerebellar segmentation mask, which was then visually inspected and manually adjusted when necessary. Next, the isolated cerebellum was normalised to the SUIT atlas template and resliced in the atlas space. Subsequently, normalized gray matter (GM) maps were modulated by scaling by the inverse of the amount of the volume changes due to spatial registration, in order to preserve the local GM amount, and then spatially smoothed using a 1 mm Full Width at Half Maximum isotropic Gaussian kernel (Smith and Nichols, 2009). The same procedure was also applied to normalized white matter (WM) maps.

Similarly to whole-brain analyses, the normalized, modulated and smoothed GM and WM maps were statistically analyzed to assess local volume differences between the two groups using a nonparametric approach based on permutations applied to the general linear model (Winkler et al., 2014) via SPM's Threshold Free Cluster Enhancement (TFCE) toolbox (http://www.neuro.uni-jena.de/tfce), including age, sex and TIV as confounding variables. 5000 permutations were generated and cluster-like structures were enhanced using the TFCE approach (Smith et al., 2006), with a significance level set at $p<0.05$, corrected for multiple comparisons across space using the FDR method ($q<0.05$) (Benjamini and Hochberg, 1995). Only those voxels were considered significant that were part of a spatially continuous

cluster size of 100 isotropic voxels or more, in order to avoid false positive results due to minor registration errors.

Results

The VBM analysis substantially confirmed the results of the whole-brain investigation, showing a broad cluster of increased GM volume in Charcot-Marie-Tooth disease type 1A (CMT1A) patients compared to healthy controls (HC) encompassing the bilateral anterior cerebellum, namely the right cerebellar lobule III (p=0.0498, d=1.80) and right (p=0.0498, d=1.44) and left (p=0.0498, d=1.58) cerebellar lobules IV and V. Smaller clusters of increased GM volume in CMT1A patients compared to HC also emerged at the level of the right cerebellar lobule VI (p=0.0498, d=1.44) and right (p=0.0498, d=1.41) and left (p=0.0498, d=1.13) cerebellar crus I (Supplementary Table and Figure 1). No significant differences emerged when testing the HC > CMT contrast regarding GM maps.

No suprathreshold clusters of altered WM quantity in CMT1A compared to HC emerged.

TABLES

Supplementary Table 1.

Clusters of increased GM volume in CMT patients compared to HC are presented, along with significance level (FDR-corrected) and the corresponding local maxima's effect sizes, T values and anatomical labels. No significant differences emerged when testing the HC > CMT contrast. Coordinates refer to mm from the anterior commissure in MNI space, with anatomical labeling according to (Tzourio-Mazoyer et al., 2002).

**Cluster Volume (ml)**	***p*-value (FDR-corr)**	**Cohen's *d***	**T**	**MNI Coordinates (mm)**			**Anatomical Label**
				**X**	**Y**	**Z**	
17.01	0.0498	1.80	5.31	15	-34	-22	Right Cerebellar Lobule III
	0.0498	1.44	4.25	15	-42	-21	Right Cerebellar Lobule IV-V
	0.0498	1.58	4.66	-23	-34	-33	Left Cerebellar Lobules IV-V
1.40	0.0498	1.44	4.27	34	-66	-20	Right Cerebellar Lobule VI
2.08	0.0498	1.41	4.18	33	-82	-36	Right Cerebellar Crus I
0.27	0.0498	1.13	3.35	-45	-63	-28	Left Cerebellar Crus I
0.47	0.0498	1.07	3.17	40	-56	-36	Right Cerebellar Crus I

DF=35

GM: Gray Matter; CMT: Charcot-Marie-Tooth; HC: Healthy Controls; FDR: False Discovery Rate; MNI: Montreal Neurological Institute; DF: Degrees of Freedom.

FIGURES

Supplementary Figure 1. Thresholded statistical maps (*in red-yellow*) for the CMT1A < HC contrast regarding GM volumes are superimposed on the SUIT T1-weighted template in axial planes.

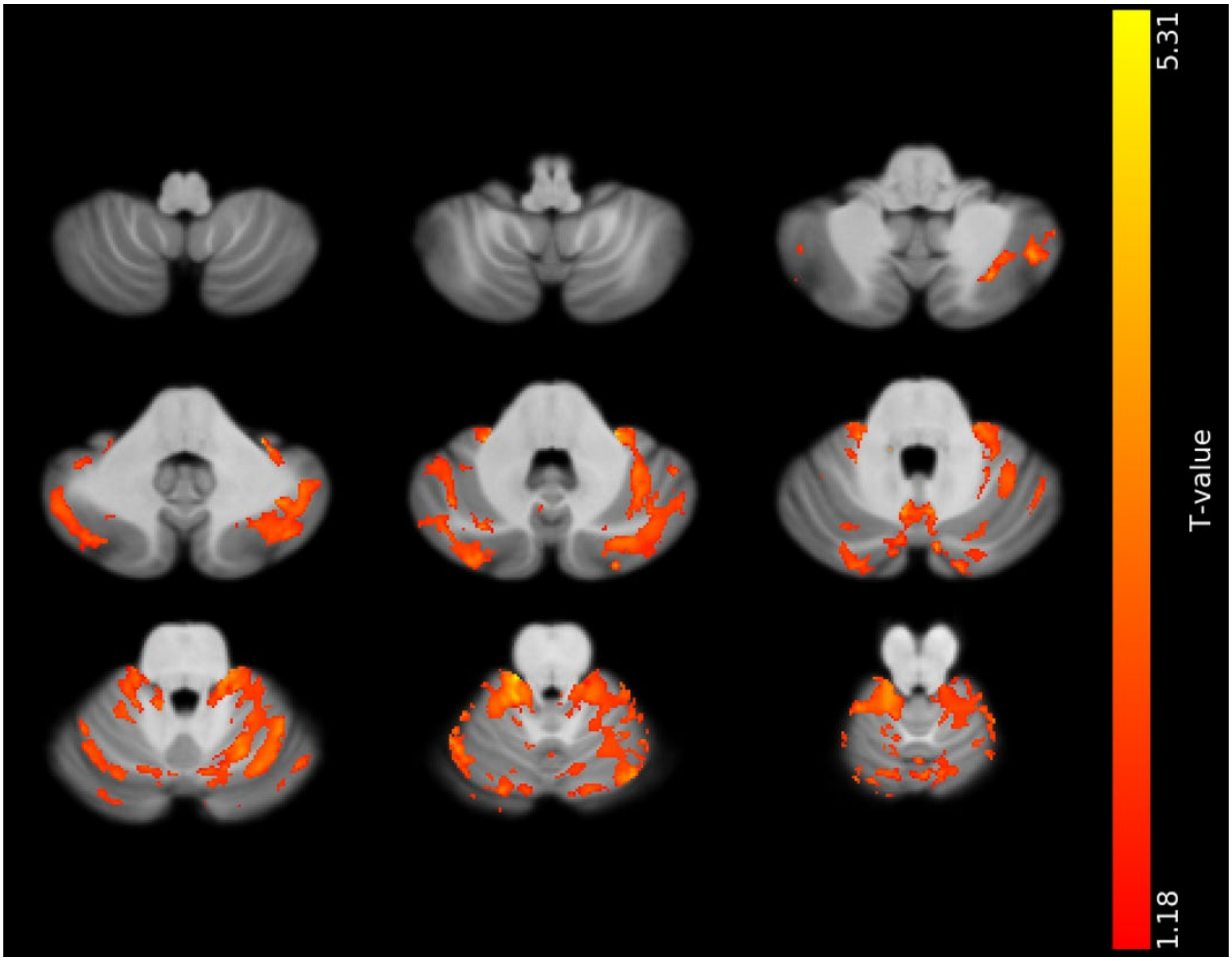

CMT: Charcot-Marie-Tooth; HC: Healthy Controls; GM: Gray Matter.

References

Benjamini, Y., and Hochberg, Y. (1995). Controlling the False Discovery Rate: A Practical and Powerful Approach to Multiple Testing. Journal of the Royal Statistical Society: Series B (Methodological) 57**,** 289-300.

Cocozza, S., Petracca, M., Mormina, E., Buyukturkoglu, K., Podranski, K., Heinig, M.M., Pontillo, G., Russo, C., Tedeschi, E., Russo, C.V., Costabile, T., Lanzillo, R., Harel, A., Klineova, S., Miller, A., Brunetti, A., Morra, V.B., Lublin, F., and Inglese, M. (2017). Cerebellar lobule atrophy and disability in progressive MS. J Neurol Neurosurg Psychiatry 88**,** 1065-1072.

Diedrichsen, J. (2006). A spatially unbiased atlas template of the human cerebellum. Neuroimage 33**,** 127-138.

Smith, S.M., Jenkinson, M., Johansen-Berg, H., Rueckert, D., Nichols, T.E., Mackay, C.E., Watkins, K.E., Ciccarelli, O., Cader, M.Z., Matthews, P.M., and Behrens, T.E. (2006). Tract-based spatial statistics: voxelwise analysis of multi-subject diffusion data. Neuroimage 31**,** 1487-1505.

Smith, S.M., and Nichols, T.E. (2009). Threshold-free cluster enhancement: addressing problems of smoothing, threshold dependence and localisation in cluster inference. Neuroimage 44**,** 83-98.

Tzourio-Mazoyer, N., Landeau, B., Papathanassiou, D., Crivello, F., Etard, O., Delcroix, N., Mazoyer, B., and Joliot, M. (2002). Automated anatomical labeling of activations in SPM using a macroscopic anatomical parcellation of the MNI MRI single-subject brain. Neuroimage 15**,** 273-289.

Winkler, A.M., Ridgway, G.R., Webster, M.A., Smith, S.M., and Nichols, T.E. (2014). Permutation inference for the general linear model. Neuroimage 92**,** 381-397.
